# Correction to “Assessment of the Trust Crisis between Upstream and Downstream States of the Helmand River Basin (1973–2022): A Half-Century of Optimism or Cynicism?”

**DOI:** 10.1021/acsestwater.4c00418

**Published:** 2024-05-21

**Authors:** Najibullah Loodin, Gabriel Eckstein, Vijay P. Singh, Rosario Sanchez

Additionally, and without consulting its upstream neighbor, Iran built four Chahnimehs, artificial humanmade reservoirs, with a total storage capacity of 1500 million cubic meters of water.^53,55,56^

